# Calcitonin estimation in patients with nodular goiter and its significance for early detection of MTC: european comments to the guidelines of the American Thyroid Association

**DOI:** 10.1186/1756-6614-6-S1-S2

**Published:** 2013-03-14

**Authors:** Rossella Elisei, Cristina Romei

**Affiliations:** 1 Department of Endocrinology and Metabolism, University of Pisa, Lungarno Pacinotti 43 - 56126 Pisa, Italy

## Abstract

One of the most discussed and controversial issue in the management of thyroid nodules is the need to perform a routine measurement of serum Calcitonin (Ct) in all cases. The American Thyroid Association guidelines do not recommend in favor or against this procedure since they retain that there are not enough evidences that it can determine an advantage to the health outcomes of these patients. This is not the view of many European experts who met in Lisbon in 2009 at the European Thyroid Association-Cancer Research Network meeting to discuss all the still open controversial issues on the management of medullary thyroid cancer patients.

This paper is focused on the routine measurement of serum Ct in all patients with thyroid nodule(s): the evidences, the rational and the benefits of this procedure are deeply analysed following the discussion that was done in Lisbon. The conclusions reached at that time are reported in detail.

## Introduction

In 2009 the European Thyroid Association endorsed the American Thyroid association guidelines for the management of medullary thyroid cancer (MTC) affected patients [1]. Although the majority of recommendations were absolutely sharable, some of them were matter of discussion because the European experts on MTC had different perspectives. At the European Thyroid Association-Cancer Research Network (ETA-CRN) Meeting held in Lisbon in 2009 an European Panel of Experts (EPE) discussed all these issues and the present paper reports in particular the EPE comments and perspectives related to the routine measurement of serum calcitonin (Ct) in patients with thyroid nodules.

## ATA recommendation 52: European comments

The ATA recommendation 52 of the guidelines for the management of MTC patients defers the recommended approach to thyroid nodules, including FNA and serum Ct testing, to the ATA guideline that addresses thyroid nodules [2]. On this regard, the recommendation 4 of the ATA guidelines for the management of thyroid nodules clearly states that the panel of American experts cannot recommend either for or against the measurement of serum Ct (recommendation rating: I). This level of rating indicates that the evidence is insufficient to recommend for or against because evidence is lacking that the intervention (i.e. serum Ct measurement in all thyroid nodules) improves important health outcomes or the evidence is of poor quality or conflicting. However, recommendation 52 of the guidelines for MTC patients management states that if a basal or stimulated serum Ct level >100 pg/ml [equal to >100 ng/L] is obtained it should be interpreted as suspicious for MTC and further evaluation and treatment should ensue (recommendation rating: A). It is worth to note that this rate of recommendation is a very strong rate since the recommendation is based on good evidence that the intervention can improve important health outcomes since the evidence includes consistent results from well-designed and well-conducted studies in representative populations. These two recommendations appear rather conflicting and in particular it is unclear how and why serum Ct should be obtained if not performed inside the diagnostic work up of a thyroid nodule.

At variance with the ATA view, the ETA consensus [3] clearly states that the routine procedure of serum Ct measurement should be applied in the work up of all thyroid nodules since it is more sensitive than fine needle aspiration cytology. It is clear that the European and American views are opposite on the regard of this issue. It is worth to note that in 2010, independently from the ETA-CRN meeting in Lisbon discussion, a new document signed by the American Association of Clinical Endocrinologists (AACE), by the Associazione Medici Endocrinologici (AME) and by the European Thyroid Association (ETA) was published which declared that routine serum Ct may be useful, especially before surgery and strongly recommended its measurement in certain high-risk group [4].

## Why is it important to measure serum Ct in nodular goiter for the EPE?

The aim of the discussion in Lisbon was to better clarify the clinical reasons and evidences to sustain the indication to perform the serum Ct routine measurement in thyroid nodules. There is no doubt that many studies demonstrated that routine measurement of serum Ct is the most accurate diagnostic tool for the detection of MTC in patients with thyroid nodules, even more sensitive than cytology [5-11]. However, low-mild elevated values of serum Ct can be either falsely positive for technical reasons [12-14] (Table 1) or for the presence of other rare pathological settings (i.e other neuroendocrine tumors, hyperparathyroidism, renal failure etc) [15] (Table 2).

**Table 1:** Technical problems in serum Ct measurement

1. Serum not appropriately stored (i.e -20°C) may give *false negative* results
2. Very high values can give *false negative* results (Hook effect)
3. Some drugs, such as omeprazole, can stimulate Ct and produce *false positive* results*
4. Heterophylic antibodies may give *false positive* results*
5. *Normal range* should be calculated in each laboratory
6. *Analytical and functional sensitivity* of the assay should be verified in each laboratory

*no positive response to stimulation with either calcium or pentagastrin

**Table 2:** Hypercalcitoninemia in pathological conditions other than MTC

1.“Small cells” lung carcinoma*
2. Various neuroendocrine tumors*
3. Chronic renal failure *
4. Pernicious anemia*
5. Zollinger’s syndrome*
6. Pancreatitis*
7. Lymphocytic thyroiditis**
8. Micropapillary thyroid carcinoma**

*no positive response to either calcium or pentagastrin stimulation

** positive response to either calcium or pentagastrin usually due to C cell hyperplasia

**Figure 1:**
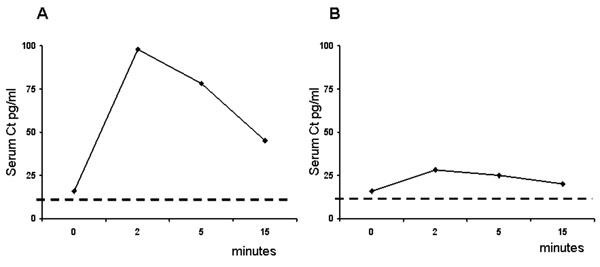
The pentagastrin stimulation test for serum calcitonin: Panel A) example of a positive stimulation test with an increase from 16 to 98 pg/ml (6 times); panel B) example of a negative stimulation test with an increase from 16 to 28 (1.7 times). The broken line identifies the upper level of our institutional normal range (i.e 10 pg/ml).

To distinguish these situations, subjects with elevated basal serum Ct should be submitted to a stimulation test which should clarify the origin of the detected Ct, especially when the basal value is low-mild elevated (i.e. between 10 and 100 pg/ml). The Ct deriving from MTC usually increases 3-4 times above the basal value after stimulation while artifactual Ct values due to technical problems or serum Ct produced by non thyroid cells usually do not increase after stimulation (Figure 1). Until few years ago the Ct stimulation test was performed with the injection of pentagastrin (Pg) (Peptavlon, Nova Laboratories, LTD, Leichester U,K 0.5 mg/kg ev) and this represented a very important limit for American colleagues because Pg was, and still is, unavailable in USA. Recently, it has been clearly demonstrated that a similar and even stronger stimulation can be obtained with a rapid infusion of calcium (2.3 mg/Kg of calcium ion or 25 mg/Kg of calcium gluconate) [16-19]. Thus, with the possibility to use calcium infusion instead of pentagastrin this limit has been overcome but it is still unresolved the problem of the stimulated Ct operative cut-off. There are several studies in which it has been attempted to clarify this issue [20-22]. However, while approaching this issue it is important to take into account that also normal subjects can have a positive response of serum Ct after stimulation but it never increases over 60 pg/ml [ng/L][17], thus a stimulated Ct between 60 and 100 pg/ml is a grey zone that deserves to be monitored. Furthermore, the rate of increase should also be considered and the clinicians should be aware that only a 3-4 time increase of stimulated Ct with respect to basal Ct should be considered as a positive response [23]. On this regard it is useful to say that these considerations are valid for sporadic cases of suspected MTC while when a hereditary case is under investigation any level of increase of either basal or stimulated Ct should be considered as positive [24].

Although the routine measurement of serum Ct in all subjects with thyroid nodules is still controversial [25,26], evidence has been provided that this approach allows an early diagnosis and treatment, thus significantly improving the outcome of this potentially lethal disease [27,28]. This evidence has been criticized by American colleagues who strongly believe that the serum Ct measurement identifies a lot of microMTC clinically irrelevant [29]. Indeed, one of the aims of measuring serum Ct in all nodules is to early identify MTC so it is conceivable that many MTC revealed by serum Ct measurement are small [30], but it is still controversial if it is true that small MTC are always good tumors [31]. Moreover, while an increase of incidence of small papillary thyroid cancer has been clearly demonstrated worldwide [32] it has never been demonstrated an increase of microMTC neither after the introduction of neck ultrasound nor in MTC series of institution adopting the Ct routine screening in thyroid nodules, which was expected if this procedure was able to bring up the hidden clinically inert microMTC.

Another very important reason to perform serum Ct measurement before surgical treatment is that if this is not done, unsuspected MTC can be unexpectedly found after thyroid lobectomy or total thyroidectomy not followed by central neck lymph node dissection that is the surgical treatment to be performed by principle when a presurgical diagnosis of MTC is available. When an incorrect surgical treatment has been already performed because the surgeon was unaware of the MTC diagnosis a personalized follow up strategy should be identified case by case by considering the serum Ct levels [33,34]. This situation is virtually impossible to be encountered in countries where the routine serum Ct measurement of all thyroid nodules is performed since even small foci of MTC not visible at neck ultrasound can be discovered by this strategy [5-8].

Last but not least is the problem of the cost-effectiveness of this screening. European studies previously demonstrated that it is valuable [22,27] but, recently, also a USA study revealed that routine Ct screening in patients with nodular goiter appeared to be cost-effective and in particular its cost-effectiveness resulted comparable to colonoscopy, and mammography screening [35].

## Conclusions

The conclusions of the EPE at the ETA-CRN meeting were that nowadays we have enough evidences to recommend routine serum Ct measurement in all patients with thyroid nodules. Moreover, it should be mandatory in all thyroid nodules patients for whom surgery has been indicated to be sure to perform the appropriate surgical treatment. However, some considerations should be always taken into account such as: a) Basal serum Ct measurement should never be performed in the absence of thyroid nodules detectable by neck ultrasound; b) Low-medium basal Ct needs to be further analysed by “stimulation”; c) Other causes of increased basal Ct should always be excluded; d) Basal Ct > 100 pg/ml must be considered very suspicious of MTC; e) A stimulated Ct < 60 pg/ml is compatible with normal response to stimulation; f) A stimulated Ct > 60 and < 100 pg/ml can be taken under evaluation by repeating the test 6-12 months later; g) The level of increasing of stimulated Ct with respect to the basal value is of great importance since in MTC it is usually greater than 3-4 times the basal value.

## List of abbreviations

Ct: calcitonin; MTC: medullary thyroid carcinoma.

## Competing interests

No competing interests exist for me and my co-authors.

## Declarations

This article has been published as part of *Thyroid Research* Volume 6 Supplement 1, 2013: European comments on Medullary Thyroid Cancer Management Guidelines of the American Thyroid Association. The full contents of the supplement are available online at http://www.thyroidresearchjournal.com/supplements/6/S1. Publication of this supplement has been supported by the European Thyroid Association-Cancer Research Network.
